# High Content Imaging (HCI) on Miniaturized Three-Dimensional (3D) Cell Cultures

**DOI:** 10.3390/bios5040768

**Published:** 2015-12-14

**Authors:** Pranav Joshi, Moo-Yeal Lee

**Affiliations:** Department of Chemical & Biomedical Engineering, Cleveland State University, 1960 East 24th Street Cleveland, Ohio, OH 44115-2214, USA; E-Mail: p.joshi18@vikes.csuohio.edu

**Keywords:** high content imaging, three-dimensional (3D) cell culture, miniaturized cell-based assay, predictive toxicology

## Abstract

High content imaging (HCI) is a multiplexed cell staining assay developed for better understanding of complex biological functions and mechanisms of drug action, and it has become an important tool for toxicity and efficacy screening of drug candidates. Conventional HCI assays have been carried out on two-dimensional (2D) cell monolayer cultures, which in turn limit predictability of drug toxicity/efficacy *in vivo*; thus, there has been an urgent need to perform HCI assays on three-dimensional (3D) cell cultures. Although 3D cell cultures better mimic *in vivo* microenvironments of human tissues and provide an in-depth understanding of the morphological and functional features of tissues, they are also limited by having relatively low throughput and thus are not amenable to high-throughput screening (HTS). One attempt of making 3D cell culture amenable for HTS is to utilize miniaturized cell culture platforms. This review aims to highlight miniaturized 3D cell culture platforms compatible with current HCI technology.

## 1. Introduction

Current high-throughput screening (HTS) technology, capable of screening a large number of new compounds against an increasing number of new targets, evaluates a single endpoint involved in drug efficacy and toxicity. However, this approach often lacks the ability to provide highly predictive information on drug responses *in vivo*, which is critical to reduce the high attrition rate in downstream drug discovery pipelines. To address this issue, high content imaging (HCI) or high content screening (HCS) technology—which refers to a high-throughput, automated microscope-based assay that provides information on multiple properties or features of individual cells simultaneously with several fluorescent dyes—has been adapted to a more systematic and accurate evaluation of drug candidates [1,2]. A tremendous amount of fluorescent cell images is quantified rapidly with image analysis algorithms to provide more predictive information on toxicity and efficacy. By measuring multiple parameters—which include target specific signals (e.g., nuclear change, organelle structure change, protein translocation, oxidative stress, apoptosis/necrosis, mitochondrial impairment, calcium homeostasis, *etc.*), reporter signal, morphology analysis, and phenotype profiling as readouts [1,3]—it is possible to understand the mechanisms of drug action and reduce the number of false positive and false negative results, which helps to identify efficacious lead compounds. Therefore, HCI has become an important tool in the drug discovery process in the pharmaceutical industry and has gained popularity for various cell-based research in academia.

Unfortunately, most current HCI assays employ cell monolayer cultured in 96-well plates (a.k.a., 2D cell cultures) for their convenience, low cost, and relatively high throughput. As compared to cultures mimicking tissues *in vivo*, these 2D cultured cells lose some of their phenotypic properties rapidly, and the formation of tissue-like structure is highly inhibited [4,5,6]. Thus, there have been enormous efforts toward developing 3D cell cultures that can maintain specific biochemical and morphological features of human cells similar to the corresponding tissues *in vivo*. Such platforms include human cells grown within the 3D structure of hydrogels or on 3D polymer scaffolds [7,8,9,10]. Scaffold-free systems such as the hanging droplet method has also been demonstrated as a high-throughput cell based assay platform [11]. These hanging droplet methods were shown to form tumor-like spheroids that partially mimic *in vivo* tumor tissue structure and have gained momentum in cancer research [11,12]. Although the hanging droplet plate has the potential to generate uniform 3D spheroids in droplets and promote cell–cell interactions, droplet spreading can be triggered by mechanical shock and surface fouling. This technique also lacks cell-extracellular matrix (ECM) interactions [13]. 

Several challenges exist in the implementation of HCI assays on a conventional 3D cell culture platform. For example, the quantitative analysis of cells present in a 3D environment in a 96-well plate is highly inconsistent and not reproducible due to the difficulty in manual handling of hydrogel and growth media [14]. Moreover, imaging and image processing poses significant challenges because cells cultured in 3D are not in a single focal plane. While such variables may not be noticeable in traditional HTS, they can become major sources of inconsistency in HCI. Furthermore, some polymer scaffolds are opaque and inadequate for imaging. When it comes to HCI, imaging technology is the key determinant of the overall success of any assay. Confocal microscopy can serve as an important tool for imaging 3D-grown cells both due to its ability to image the cells at high resolution in different optical sections and integrate the sectioned images [15]. However, slow point scanning of confocal microscopy induces low throughput of image acquisition, which can be problematic for large-scale screening and may incur some photobleaching and phototoxicity [16,17]. Light-sheet microscopy is an alternative, promising technology for HCI due to its ability to image biological samples in 3D for longer time without damaging the cell samples. However, implementing this technology in a core facility requires complete changes in experimental methods being used, and the commercial systems are still not fully accessible [18]. Moreover, an enormous amount of data generated (terabytes of data per day) further limits the implementation of this technology in standard facilities, dealing with gigabytes of data [17]. 

Miniaturized 3D cell culture technology, thus, can be a better choice for those who do not want to compromise throughput yet want to have better imaging features required for HCI. In contrast to conventional macroscale 3D cell culture such as in 96-well plates, miniaturized 3D cell culture allows the whole sample depth to fit within the focus depth of a normal objective due to its small dimension. Additionally, miniaturization of 3D cell culture allows for high control over microenvironmental cues, enabling more reproducible outcomes [19]. Finally, miniaturization can reduce reagent consumption, easily facilitate combinatorial approaches, and minimize the use of valuable materials, such as patient-derived cells [13,14].

This article aims to summarize existing miniaturized 3D cell culture systems that have great potential in HCI applications. HCI assays and some of their major applications are described in the first section. Importance of 3D cell culture technology in HCI and the limitations of traditional 3D cell culture systems are highlighted in the second section. Finally, current miniaturized cell-based assay systems that have demonstrated HCI capabilities are discussed, along with potential challenges in implementing HCI assays on miniaturized 3D cell culture systems.

## 2. High Content imaging (HCI) Assays and Their Applications

HCI assays can probe a myriad of cellular processes at the individual cell level, including cell growth, cell viability/cytotoxicity, changes in nuclear function, apoptosis/necrosis, mitochondrial membrane potential (MMP), oxidative stress, intracellular calcium levels, and glutathione levels [20,21,22]. Target- and phenotype-based HCI assays are expected to provide multi-parametric information on cellular functions and processes that play pivotal roles in human toxicology. By investigating specific cellular functions at the individual cell level, one can analyze potentially heterogeneous cell populations within microenvironments of a tissue that have different properties due to oxygen/nutrient/compound diffusion limitations, or cell populations that are impacted by the micro-heterogeneous nature of ECMs. HCI assays have been implemented using various cell types such as primary cells [22], immortalized cell lines [20], and stem cells [23], for applications ranging from investigating the toxicity of nanoparticles [24] to investigating cardiotoxicity [21,25] and neurotoxicity [26,27]. HCI assays are performed using a variety of fluorescent probes, including Hoechst 33342 for nuclear morphology and cell count, calcein AM and propidium iodide (PI) for cell viability, tetramethyl rhodamine methyl ester (TMRM) for mitochondrial membrane potential (MMP), Fluo-4 AM for intracellular calcium levels, YO-PRO-1 for apoptosis, monochlorobimane (MCB) for glutathione levels, and 2',7'-dichlorodihydrofluorescein diacetate (H_2_DCFDA) for oxidative stress damage (Table 1). Additionally, a suite of HCI assays have been developed to assess mechanisms of compound toxicity and evaluate the effects on organ toxicities, such as hepatotoxicity, cardiotoxicity, and neurotoxicity (Table 2).

**Table 1:** Commonly used fluorescent probes for various high content imaging (HCI) assays.

Assay/ Endpoint	Target Organelle	Fluorescent Probe	Color	Excitation/Emission (nm)	References
Nuclear morphology/ Cell number	Nucleus	Hoechst 33342	Blue	361/497	[20,28,29,30,31,32]
	Nucleus	Hoechst 33258	Blue	352/461	[23,27]
	Nucleus	Draq5	Red	647/681	[33]
	Nucleus	DAPI	Blue	350/470	[34]
Cell viability	Cytoplasm	Propidium iodide	Red	535/620	[20,29]
	Cytoplasm	Calcein AM	Green	495/520	[23]
Cell membrane permeability	Nucleus	TO-PRO-3	Red	642/661	[16,19]
	Nucleus	BOBO-1	Green	462/481	[21]
Apoptosis	Nucleus	YO-PRO-1	Green	490/510	[35]
	Caspase 3	Anti-caspase 3 antibody*	*	*	[21,36]
	Mitochondria	Anti-cytochrome C antibody*	*	*	[36]
Mitochondrial membrane potential	Mitochondria	TMRM	Red-Orange	545/575	[20,24,29]
	Mitochondria	MitoTracker	Orange	554/576	[23]
Intracellular calcium level	Calcium ions in cytoplasm	Fluo-4 AM	Green	490/520	[20,24]
Glutathione level	Glutathione in cytoplasm	MCB	Blue	380/460	[22,28]
Reactive Oxygen Species (ROS) generation	Oxygen radicals in cytoplasm	BODIPY 665/676	Red	665/676	[20]
	Oxygen radicals in cytoplasm	H2DCFDA	Green	495/527	[29]
Lipid accumulation	Lipids	BODIPY 493/503	Green	493/503	[29]
Cell cycle disruption	Nucleus	Anti-phospho histone H3 antibody*	*	*	[32,34,36]
	Nucleus	EdU	Green	495/519	[32,36]
Lyososomal acidification	Lysosome	LysoTracker	Green	504/511	[24]

Abbreviations are used as follows: 4',6-diamidino-2-phenylindole (DAPI), fluo-4 acetoxymethyl ester (fluo-4 AM), tetramethyl rhodamine methyl ester (TMRM), monochlorobimane (MCB), 2',7'-dichlorodihydrofluorescein diacetate (H2DCFDA), 5-ethynyl-2'-deoxyuridine (EdU). * Color and excitation/emission wavelengths are changed depending on secondary antibodies conjugated with primary antibodies.

**Table 2:** Multiple parameters used in HCI assays and their applications in various areas of research.

Research Areas	Applications	HCI Assays	References
**Toxicology**	Screening of compounds for cytotoxicity	Apoptosis, necrosis, and measurement of cell numbers and morphological features	[34]
	Hepatotoxicity screening with HepaRG cells	Cell count, nuclear size, and in-cell CYP3A4 expression	[28]
	Hepatotoxicity screening with iPSC-derived hepatocytes	Cell viability, cell shape, cell area, nuclear shape, mitochondria potential, autophagy, and phospholipidosis	[23]
	Identification of drugs inducing steatosis	Lipid content, ROS generation, MMP, cell viability, and cell count	[29]
	Hepatotoxicity screening and mechanisms of drug action	Cell viability, nuclear morphology, lipid peroxidation, MMP, and intracellular calcium concentration	[20]
	Cardiotoxicity screening with stem cell-derived cardiomyocytes	Nuclear morphology, MMP, apoptosis, and cell membrane permeability	[21]
	Developmental neurotoxicity with neurons	Quantification of βIII-tubulin (neurite marker), pNF (axonal marker), and MAP2 (dendrites marker)	[27]
	Mechanism of drug action for inhibiting tumor cell growth	Apoptosis, cell cycle disruption, DNA damage, and cellular morphology	[36]
	Developmental neurotoxicity	Metabolic activity with resazurin, nuclear morphology, neurite outgrowth, and cell viability	[26]
**Nanotoxicology**	Cytotoxicity of amine-modified polystyrene nanoparticles	Nuclear morphology, MMP, cytosolic calcium, lysosomal acidification, and plasma membrane permeability	[24]
**Cancer**	Inhibition of STAT3 pathways in head and neck cancer	Nuclear morphology and pSTAT3-Y705 staining	[30]
	Identification of phage antibodies that bind to tumor cells via macro pinocytosis	Detection of cell-associated IgG, cell-associated phage, and nuclei	[31]
	Up-regulation of Pfn-1 in metastatic breast cancer	Cell migration, chromatin condensation, cell density, cell size, nucleus area, actin content, and actin fiber	[37]
**Infectious Disease**	Cell cycle arrest by Ebola virus infection	Quantification of cells in S-phase and M-phase, nuclear size, and nuclear intensity	[32]
	Screening of protease-inhibiting compounds against rift valley fever virus	Detection of Gn antibody staining, nuclear and cytoplasmic intensities of G signal, nuclear size, and nuclear intensity	[38]
	Burkholderia pseudomallei (Bp)-induced formation of multinucleated giant cells in murine macrophages	Cell number, area, number of bacterial spots, and anti-Bp antibody staining	[39]
	Screening of compounds against Chagas disease	Number of nuclei, amastigotes, and percentage of infected cells per well	[33]
	Identification of Coxiella burnetii bacterial factors involved in host cell interaction	Nuclei number, fragmentation, area, perimeter, GFP intensity of coxiella colonies	[40]
**Epigenetics**	Identification of JMJD3 chemotypes to understand the role of demethylase	Quantification of JMJD3 expression and histone H3-specific antibody staining	[41]
**Neurodegenerative Disorder**	Identification of drugs for Huntington’s disease	Number of somata, area of somata, neurite length, and neurite area	[42]

Abbreviations are used as follows: induced pluripotent stem cell (iPSC), cytochrome P450 3A4 (CYP3A4), Pan axonal neurofilament (pNF), microtubule associated protein 2 (MAP2), signal transducer and activator of transcription 3 (STAT3), profilin 1 (Pfn-1), envelope glycoprotein (Gn), immunoglobulin G (IgG).

The application of HCI assays has been widely reported in several areas of research. However, due to the limited scope of our review, we would like to discuss few major applications of HCI, including toxicology and cancer research. The application of HCI in toxicology such as the investigation of the hepatotoxic potential of drugs is reported by several groups [15,17,23,43]. For example, Tolosa *et al.* measured various endpoints of drug-induced hepatotoxicity in HepG2 cells including nuclear morphology, MMP, cell viability, intracellular calcium level, and oxidative stress and evaluated hepatotoxicity of seventy-eight compounds with known mechanisms of action [20]. However, metabolism-induced hepatotoxicity has not been demonstrated because of metabolically incompetent HepG2 cells used [20]. To improve the predictability of HCI assays on metabolism-mediated toxicity, hepatic cell lines were infected with recombinant adenoviruses carrying genes for cytochrome P450 (CYP450) isoforms to transiently express drug metabolizing enzymes (DMEs) [44]. Recently, Ranade *et al.* demonstrated a HCI assay with a DME-expressing hepatic cell line such as HepaRG cells for hepatotoxicity screening [28]. Various toxicity parameters such as glutathione level, MMP, cytoskeletal change, and cell viability have been measured along with the expression level of CYP450s in HepaRG cells to demonstrate the metabolic competency of the cell line and relevant hepatotoxicity of metabolically competent and incompetent cells [28]. Hepatocytes derived from human induced pluripotent stem cells (hiPSCs) have been used to study general cytotoxicity and mechanism-induced hepatotoxicity of compounds by characterizing cellular and nuclear morphology, lipid accumulation, and MMP [23]. HiPSC-derived hepatocytes expressed various hepatic biomarkers, including lipid accumulation, tight junction formation, and glycogen storage ability comparable to primary hepatocytes, indicating an increased sensitivity and specificity of the assay when using hiPSCs [23]. Similarly, cardiotoxicity has been investigated with hiPSC-derived cardiomyocytes by evaluating apoptosis, MMP, nuclear morphology, and cell membrane permeability [21]. Additionally, HCI assays have been successfully implemented for the study of neurotoxic compounds in primary neurons [27] and immortalized neuronal precursor cells [45]. Quantification of neurite outgrowth, nuclear morphology, cell viability, and other axonal and dendritic markers are evaluated to study the effect of drugs on developing and mature neurons [26,27].

In addition to toxicity assays, HCI assays have been implemented to detect pathogens inside cells and to identify drugs against those pathogens [40,46]. The ability to characterize cellular phenotypes in response to bacterial and viral infections make HCI assays powerful tools in infectious disease research, including Chagas disease [33], Hepatitis C [47], and Ebola [32]. For example, Alonso-Padilla *et al.* evaluated the effect of potential drugs on *Trypanosoma cruzi* parasite (Chagas disease) and the toxicity on host cells simultaneously by measuring the number of nuclei, the number of amastigotes per cell, and the percentage of infected cells per well [33]. Efficacy of drugs against other lethal viral pathogens such as Ebola [32] and Rift Valley fever virus (RVFV) [38] have also been evaluated by measuring nuclear size, nuclear intensity, cell number, and cell area.

Moreover, HCI assays have been widely used in cancer research to study the changes of morphological and functional features such as motility, growth, proliferation, and death by anticancer drugs [36,37]. For example, Towne *et al.* investigated the mechanisms of tumor cell death such as apoptosis, cell cycle disruption, and DNA damage by a panel of HCI assays [36]. Activation of caspase 3 and cytochrome C release for apoptosis, phospho-histone H3 and DNA synthesis for cell cycle disruption, and phospho-histone H2AX for DNA damage were measured along with cell morphology and nuclear swelling [36]. Potential inhibitors of cell motility in metastatic breast cancers have been identified by measuring cell migration, cellular and nuclear morphology, and actin depolymerization [37]. Inhibitors of oncogene signaling pathways such as STAT3 involved in development and progression of head and neck squamous cell carcinomas (HNSCC) have been screened by measuring nuclear morphology and anti-pSTAT3-Y705 antibody staining [30]. Apart from identifying compounds inhibiting cancer cell motility, growth, and progression, HCI assays have been applied to investigate drug-antibody conjugates that can bind specifically to cancer cells and deliver a drug to cellular targets [31]. In addition, it has been used to screen a library of photodynamic therapy (PDT) compounds for cancer treatment [48]. 

## 3. Macroscale Three-Dimensional (3D) Cell Cultures Applicable to HCI

**Figure 1:**
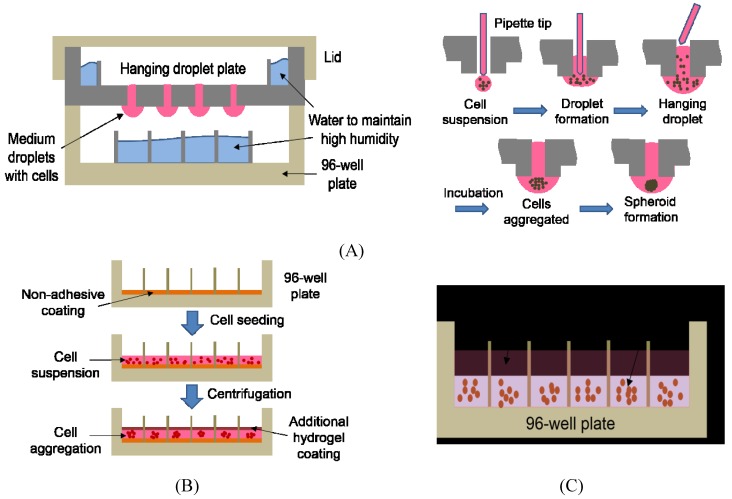
Commonly used 3D cell culture techniques for HCI. (**A**) Hanging droplet plate containing 3D spheroids. To generate 3D spheroids, cell suspension is dispensed through the access holes of the hanging droplet plate such that the droplets are attached to the hydrophilic surface. Individual cells are aggregated within hours of incubation due to gravity, forming a single spheroid. (Adapted from [57] with permission of The Royal Society of Chemistry.) (**B**) Liquid overlay on top of cells in a 96-well plate. The bottom of the 96-wells is coated with non-adhesive polymer in a serum-free medium, which is followed by cell seeding. The 96-well plate is centrifuged to induce cell aggregation and hydrogel in a serum-supplemented medium is overlaid on top of the aggregated cells. (**C**) Hydrogel matrix mixed with cells in a 96-well plate. Cell suspension is mixed with hydrogel and dispensed in the 96-well plate. Cells naturally form 3D structures within the hydrogel matrix while growing.

Cells cultured in conventional 2D monolayer system vary significantly from the 3D-cultured cells in terms of their properties such as morphology, physiology, protein/gene expression, and metabolism [4,9,49,50]. Cells grown in 2D monolayer have limited intercellular contacts and interactions because the formation of tissue-like structure is inhibited and some of their phenotypic properties are also lost [4,5,14,51]. The importance of 3D cell culture in maintaining normal cell function such as differentiation, migration, and proliferation has been highlighted in various literatures [52,53,54,55]. Moreover, important biological cues are provided to cells by the ECM in response to external stimuli [49,52]. Therefore, various 3D cell cultures have been carried out using techniques such as hydrogel matrices [40,41], 3D hanging droplets [42,43], and liquid overlay technique [55,56] among others (Figure 1 and Table 3). Due to the limited scope of our review, we focus on 3D cell culture technologies that can be applicable to HCI. For more general information on 3D cell culture technologies, readers are recommended to read other review papers written by Montanez-Sauri *et al.* [13], Page *et al.* [49], and Justice *et al.* [51]. Since cell behavior and characteristics are heavily influenced by growth conditions, they are more likely to mimic *in vivo* characteristics in a 3D model rather than in a 2D model.

**Table 3:** Advantages and disadvantages of commonly used 3D cell culture techniques.

Cell Cultures	Advantages	Disadvantages	Applications (References)
**Hydrogel Matrix**	Cell–ECM interactions, easy to incorporate growth factors, *in vivo*-like microenvironments, long-term culture, uniform spheroid	Cumbersome to dispense cells in hydrogels and change growth media, thus low throughput, difficult to retrieve cells after 3D formation	*In vitro* angiogenesis and drug testing [57,58]; Drug response study [14,59]; Cancer research [60]
**Hanging Droplet**	Simple spheroid formation by gravity, homogenous spheroids that are easily accessible	Labor intensive and time consuming, no cell-ECM interaction, difficult to change growth media, transferring of spheroids for analysis required, sensitive to mechanical shocks	Hepatotoxicity testing with HepaRG cells [61,62]; Target identification and validation using RNAi [63]
**Liquid Overlay**	Simple to use, inexpensive, long-term culture	Labor intensive and time consuming, low throughput due to the centrifugation step involved, heterogeneous spheroids, difficult to mass produce	Evaluation of therapeutic response of anticancer drugs [58]; Identification of anticancer drugs [55]; Hepatotoxicity testing with iPSC-derived hepatocytes [64]

3D cell culture technology has found a niche in toxicology research due to the enhanced functionality of cellular models [62]. For example, maintenance of long-term liver-specific function and high predictability towards drug-induced hepatotoxicity have been demonstrated in several studies with 3D cell models [61,62]. Gunness *et al.* reported the expression of liver-specific proteins including albumin, phase I DMEs (CYP2E1 and CYPA4), and efflux transporter (MRP-2) in HepaRG cells cultured in 3D by the hanging droplet technique, which resulted in high predictability of hepatotoxicity of tested drugs [61]. Similarly, Mueller *et al.* demonstrated the long-term liver-specific functionality of 3D-cultured HepaRG cells with significant levels of expression of CYP3A4 and MRP-2 and production of glucose and lactate [62]. More recent work on hepatotoxicity has shown 3D culture of hepatocytes differentiated from hiPSCs to be an effective platform for drug toxicity testing, with high levels of phase I and phase II DME expression along with high levels of albumin and urea secretion and expression of various drug transporters [64]. These hepatocytes differentiated from iPSCs were shown to have greater sensitivity in predicting drug-induced hepatotoxicity along with reactive metabolite-mediated toxicity of twenty-five test compounds [64].

Significant changes in cellular morphologies and responses have been observed in primary hepatocytes and human hepatoma cell lines cultured in 2D *versus* 3D environments. Three-dimensional cultures of primary hepatocytes and hepatoma cell lines have been shown to exhibit liver-specific functions such as secretion of urea and albumin and expression of phase I and phase II DMEs, whereas cells cultured in 2D monolayer were shown to lose their liver-specific functions [65,66,67]. Moreover, cells cultured in 2D and 3D exhibit differential responses towards drugs as the 3D-cultured cells showed an increased chemoresistance to anticancer drugs [68,69,70] attributing to its multicellular drug resistance (MDR) characteristic [71].

Increased physiological relevance of 3D cell culture for toxicity testing is highly evident. However, very little effort has been put towards the miniaturization of existing 3D cell culture systems that are compatible with large-scale HCI [55,56]. The establishment of more predictive 3D cell-based screens for drug efficacy and toxicity testing requires both the development of high-throughput platforms compatible with automated robotic systems that enable rapid and reproducible testing of 3D cultures, and a fundamental understanding of the mechanisms that drive the differential response of the 2D and 3D cultures to various stimuli and environmental factors. This is a challenging task that involves the decoupling of variables such as structural organization of cells, cell-cell and cell-matrix interactions, mechanical and biochemical cues, cell density, and nutrient and drug penetration. HCI assays can play a significant role in overcoming these challenges and provide highly predictive outcomes offered by 3D cell models.

Incorporating HCI assays in 3D cell culture (3D HCI) helps us to decipher the mechanisms of *in vivo* toxicity by compounds and provides better understanding of various adverse reactions by human tissues. However, very few HCI assays have been demonstrated in 3D cell models, with the majority of them being carried out in 3D spheroid models of tumor cells for identifying anticancer drugs [72,73]. For example, Krausz *et al.* demonstrated the feasibility of screening anticancer drugs using 3D multicellular tumor spheroids by evaluating the size of cell colony [72]. Wenzel *et al.* identified compounds targeting inner non-proliferative tumor cells, where spheroids were identified by nuclei staining, and cell proliferation, viability, and apoptosis induction were quantified [73]. In addition, Reid *et al.* developed a 3D HCI platform to identify inhibitors of cytokeratin 5 (CK5), a biomarker of breast cancer cells, by quantifying changes in the expression level of CK5 promoter-enhanced green fluorescent protein (CK5Pro-GFP) with intensity sum and area parameters [74].

HCI assays on macroscale 3D culture systems pose inherent challenges as acquisition of 3D-grown cell images is highly restricted by light scattering due to the thickness of cellular models and impaired diffusion of reagents across multiple layers of cells [75,76]. Optical clearing protocols are often implemented to improve the imaging capability of 3D cell structure [76,77,78]. However, optical clearing agents used are often cytotoxic and may incur changes in cell and tissue morphology, thereby limiting the application of optical clearing protocols [75]. Throughput is another important factor when it comes to implementing 3D HCI assays in large-scale drug efficacy and toxicity testing. Imaging macroscale 3D cell models takes a longer time due to Z-focus position issues, and 3D-cultured cell images acquired are often inconsistent even within the same 96-well. In addition, conventional 3D cell culture systems that require relatively large assay volumes are not amenable to HTS mainly due to difficulty in handling viscous solutions. Dispensing the mixture of cell suspension and viscous hydrogel and changing growth media over time without affecting the consistency and reproducibility in 96-well plates are a challenging task [14]. Moreover, the cost of reagents and compounds in conventional 3D culture systems possesses limitation in the number of assays that can be performed for HCI [13]. Given the limitations of macroscale 3D cell culture systems, very few 3D HCI assays have been implemented. To address these issues, miniaturized 3D cell culture systems with high-throughput, HCI capability have been studied.

## 4. Miniaturized 3D Cell Culture Systems and Their Application in HCI

**Figure 2:**
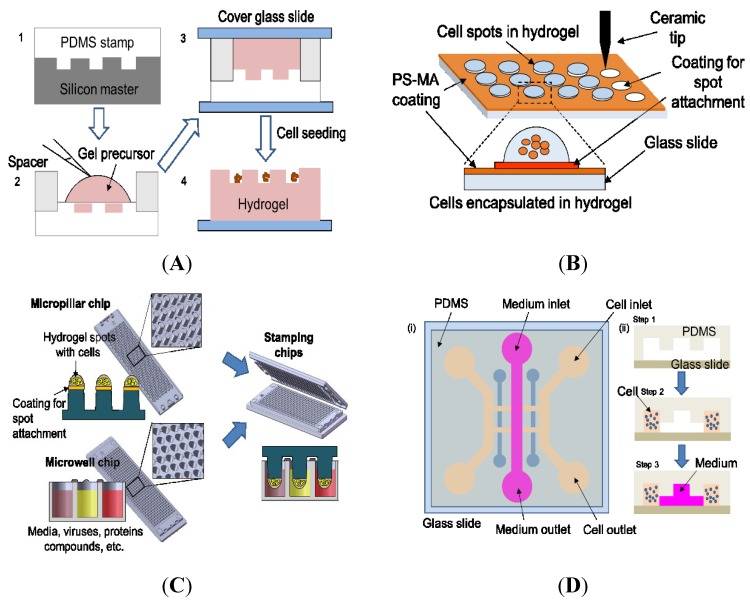
Miniaturized 3D cell culture systems for HCI. (**A**) **Microwell platform** (Adapted from Ref [90] with permission of The Royal Society of Chemistry). Overview of hydrogel microwell arrays fabrication process: (Step 1) A polydimethylsiloxane (PDMS) stamp containing an array of micropillars is cast on a silicon master. (Steps 2 and 3) Poly(ethylene glycol) (PEG) gel is cross-linked to contain complementary microwell array topography using the PDMS stamp as a template. (Step 4) Individual cells are trapped on the hydrogel surface after swelling and washing of the surface. (**B**) **Cellular microarrays** **on a functionalized glass slide**. A mixture of cells and hydrogel precursor is printed on a glass slide coated with poly(styrene-co-maleic anhydride) (PS-MA). Various polymer coating is done on top of the PS-MA coating to attach different hydrogels to the glass slide. Cells are encapsulated in a hydrogel matrix, forming 3D structures after gelation (which occurs via various mechanisms). (**C**) **Cellular microarrays on a micropillar/microwell chip platform** (Adapted by permission from Macmillan Publishers Ltd: Nature Communications, Ref [84]). Cells mixed with hydrogel are printed on top of the micropillar chip. After gelation, the micropillar chip containing cells encapsulated in hydrogel is sandwiched with a complementary microwell chip containing growth media or other reagents. (**D**) **Microfluidic** **device**. (i) Top view of a bilayer microfluidic chip fabricated with PDMS on top of a glass slide. Several inlet and outlet channels provide parallel access to cell suspension, growth medium and other reagents. (ii) Overview of the cell culture process in the microfluidic device: (Step 1) Bi-layer chip is fabricated with PDMS containing several channels on top of a glass slide. (Step 2) A mixture of cells and hydrogel precursor is fed from the cell inlet channel. (Step 3) A growth medium is supplied from the medium inlet channel for cell culture.

Conventional macroscale 3D cell culture systems are not well suited to rapidly investigate the complex *in vivo*-like 3D microenvironments due to aforementioned limitations in imaging and HTS capability [19,76,79]. On the other hand, miniaturized 3D cell models significantly reduce assay volume and reagent consumption and provide excellent control over cellular microenvironments by precisely managing cell culture conditions in a combinatorial fashion within small dimensions. Due to the reduced sample volume required, we may be able to use expensive and scarce patient-derived cells, which may lead to enhanced predictability of *in vivo* drug responses in individuals [19]. Miniaturization of 3D cell models can reduce the time required for image acquisition and analysis, which makes 3D cell culture system more amenable to high-throughput HCI. Image acquisition would be much simpler due to the thin depth of Z-focus position of samples, leading to significantly increased signal-to-noise ratio [80]. Current miniaturized 3D cell culture models can be broadly classified into three categories: microwell platforms, cellular microarrays, and microfluidic devices (Figure 2). The following section summarizes these miniaturized 3D cell culture platforms and highlights several HCI assays demonstrated on these platforms (Table 4).

**Table 4:** Advantages and disadvantages of miniaturized 3D cell-based assay systems.

Miniaturized 3D Culture Systems	Advantages	Disadvantages	Applications (References)
**Microwell platform**	Control over spheroid size, HCI compatible	Cumbersome to fabricate microwells manually, less work done with ECMs, difficult to test compounds in each microwell due to well-to-well cross contamination, low throughput	Study of self-renewal and differentiation of stem cell [81]; Study of cancer and drug development [82]
**Cellular microarray**	Easy to add compounds and biomaterials, cell-ECM interactions allowable, high throughput, HCI compatible	Optimization required to prevent spot detachment, temperature and humidity control required to minimize evaporation, relatively short-term culture	Metabolism-induced toxicity [83,84]; HTS of anti-cancer drug efficacy [85]; Quantification of protein levels [86]; Study of drug toxicity screening [87]; Evaluation of ajoene toxicity *in vitro* [88]
**Microfluidic device**	Possible to test chemical gradients, control of fluids and cell locations to specific regions, HCI compatible	Cumbersome fabrication of microfluidic devices required, low throughput due to manual intervention and bulky pumps, bubble formation, channel clogging by cells	Drug-induced cardiotoxicity screening [25]; Analysis of ECM interaction and response to external stimuli [89]

### 4.1. Microwells

The microwell platform consists of thousands of wells with various shapes that are fabricated using photolithography techniques; this platform can be used for miniaturized 3D culture of individual or groups of cells [91]. Microwells are fabricated with non-adhesive, biocompatible materials such as polyethylene glycol (PEG), polydimethylsiloxane (PDMS), polyacrylamide, chitosan, or agarose [79,92]. Cell suspension is dispensed on the non-adhesive microwells, allowing them to settle via gravity (Figure 2A). Uniform multicellular tumor spheroid formation has been demonstrated with the ability to control the spheroid diameter in microwells for HTS of drugs [93]. Accurate control over culture conditions within microwell platforms have been explored for the study of extrinsic regulators of stem cell fate [81] and the study of drug responses in cancer cells [82]. For example, Hakanson *et al.* developed PEG microwells with tunable protein coating that can provide increased control over cell culture, cell-cell, and cell-matrix interactions [82]. HCI assays such as nuclei density, nuclei morphology, cell proliferation, and apoptosis were demonstrated on this platform to study drug responses against cancer cells [82]. In addition, HCI assays have been demonstrated in PDMS microwells with immune cells that were monitored in real-time to evaluate cell viability, MMP, reactive oxygen species (ROS) generation, and plasma membrane integrity for the cytotoxic effect of drugs [94]. Major limitations of the microwell systems are the potential for cross-contamination between microwells, the difficulties in dispensing multiple drugs directly into the microwells and monitoring drug responses, and the requirement of expensive and time-consuming fabrication steps.

### 4.2. Cellular Microarrays

The cellular microarray platform consists of 3D cell spots encapsulated in a hydrogel matrix on glass slides or plastic chips (Figure 2B,C). A mixture of cells and hydrogel precursor is printed onto functionalized glass slides or micropillar/microwell chips using microarray spotters, which can form gels by various methods such as temperature change, ionic cross-linking, UV irradiation and so on [83,85]. The cellular microarrays have been applied to various cell-based assays, including the study of cell-ECM interactions for cell-adhesion profiling [95], the testing of drug candidates and their metabolites for metabolism-induced toxicity [83,84], the quantification of protein levels in cells [86], stem cell differentiation and toxicity [96,97], and HTS of anticancer drug efficacy [85]. Recently, a micropillar chip and a complementary microwell chip have been developed for high-throughput cell-based assays such as 3D cultures of mammalian cells, enzymatic reactions, viral infection, and compound screening [84,85]. The micropillar chip, made of functional poly (styrene-*co*-maleic anhydride) (PS-MA), supports 3D cell cultures and comprises an array of human cells for gene expression and toxicity screening (Figure 2C). 

The cellular microarray technology on the chip offers several advantages over conventional 3D cell culture approaches in cell-based assays. Specifically, it requires extremely small amounts of cells, natural and synthetic hydrogels, ECMs, growth factors, compounds, and other reagents for creating and evaluating 3D cell cultures. Cell encapsulation protocols developed are flexible and allow for culturing multiple cell types from different tissues in hydrogel layers on the chip, consequently providing more insight into potential tissue-specific toxicity of compounds [85,87]. Miniaturized 3D cell cultures on the chip may provide a microenvironment that simulates the *in vivo* ECM conditions, and therefore help to maintain the specific biochemical functions and morphological features of human tissues similar to those found *in vivo*. In addition, gene transduction protocols established on the chip can be applied to miniaturized 3D cell cultures to study gain- and loss-of-function genomic screening in oncology. For example, Fernandes *et al.* in their earlier work demonstrated the capability of HCI assays in cellular microarrays with fluorescent probes for cell viability and immunofluorescence staining and quantified the α subunit of hypoxia-inducible factor (HIF-1α) proteins in human pancreatic tumor cells [86]. HCI of 3D cell cultures on the chip is in early stages of research, and has yet to be fully applied towards toxicology. One of the major limitations of cellular microarrays is the spot detachment issue (*i.e.*, the detachment of hydrogel/cell spot from the pillar chip due to poor interaction between PS–MA surface and the hydrogel used). Sophisticated surface chemistry is required to prevent the spots from getting detached from the chip surface. In addition, it is not suited for long-term cell culture due to the small dimension of cell spots. 

### 4.3. Microfluidic Devices

A microfluidic device is a compact, monolithic bi-layer chip fabricated with PDMS that contains an array of microchannels and chambers for cell cultures, and inlets and outlets for providing access to desired reagents (Figure 2D) [98,99]. The advantage of microfluidic device for culturing cells under various flow conditions in a single chip has been utilized in HCI assays for toxicity tests [100]. For example, Ye *et al.* developed an integrated microfluidic device consisting of multiple concentration gradient generators (CGGs) and parallel cell culture chambers, and demonstrated the capability of HCI assays by screening various concentrations of anticancer drugs for induction of apoptosis in HepG2 cells [101]. Similarly, Yu *et al.* demonstrated the capability of parallel processing on the microfluidic device by measuring the apoptotic effect of drugs on NIH-3T3 fibroblasts, B16 melanoma, and HeLa cell lines [89]. Apart from drug toxicity screening, microfluidic devices have been utilized in various cell-signaling studies [99,102]. Cheong *et al.* developed an immunofluorescence assay for the quantification of individual cell signaling networks using a microfluidic device and demonstrated the HCI capability by measuring the signaling activity of kinases, nuclear factor kappa-light-chain-enhancer of activated B cells (NF-kB), and other target genes in NIH-3T3 fibroblasts in response to stimulation from cytokines and chemical inhibitors [99]. Comprehensive information on HCI assays performed in microfluidic devices can be found in the review by Cheong *et al.* [103]. Additionally, the production of multicellular spheroids in microfluidic devices has been recently demonstrated in areas such as anticancer therapies [104,105,106] and tissue engineering [107]. One of the major limitations of microfluidic devices is its relatively low throughput in terms of cell-based assays. Moreover, issues such as absorption of hydrophobic molecules, needed for frequent media change, and PDMS toxicity impose further limitations in 3D HCI [108,109]. 

## 5. Conclusions

High-throughput HCI on miniaturized 3D cell culture has a potential to decipher toxicodynamic and toxicokinetic traits of drugs and helps us to understand complicated toxicology pathways and related adverse responses in early stages of drug discovery. Simplifying steps for miniaturized 3D cell culture and integrating the cell culture platforms with automation systems for liquid handling and image acquisition/analysis are critical to implement 3D HCI. Microwell and cellular microarray platforms are more compatible with automated liquid dispensing robots such as microarray spotters than microfluidic devices, resulting in increased throughput of HCI, whereas microfluidic devices are inherently low throughput in HCI due to the sample loading processes and large pumping units [19]. In addition, commercially available HCI-imaging systems such as Cellomics and GenePix scanners that have been developed for cells cultured on 2D surfaces may not be suited for cells cultured in 3D platforms due to Z-focus positioning and incompatibility with platform dimensions [13]. Throughput of HCI assays can be highly compromised due to time-consuming confocal imaging. In this regard, light-sheet microscopy can be a better option as it provides high-throughput 3D imaging capability without damaging cell samples. Commercial light-sheet microscopy systems are available although users may need to wait for some more time to get fully acquainted with this technology [16,18]. Needless to say, there currently is no single solution for all the imaging requirements. Various assays and applications will certainly require some level of customization by the user utilizing any imaging system. Finally, more effort will be required to generate physiologically relevant 3D cell models while maintaining the high-throughput capability of cell-based assays. 
